# Preoperative phase angle and postoperative complications in Crohn’s disease patients undergoing ileocolic resection: a retrospective cohort study

**DOI:** 10.1007/s00384-025-05053-y

**Published:** 2026-01-03

**Authors:** Weilin Qi, Huaying Liu, Huiping Liang, Wei Liu, Linna Ye, Qian Cao, Xiaolong Ge, Wei Zhou

**Affiliations:** 1 Department of General Surgery, School of Medicine, Sir Run Run Shaw Hospital, Zhejiang University, 3 East Qingchun Road, Hangzhou, 310016 China; 2 Department of Medicine, Guangxi Health Science College, Nanning, China; 3 Department of Gastroenterology, School of Medicine, Sir Run Run Shaw Hospital, Zhejiang University, Hangzhou, China; 4 Inflammatory Bowel Disease Center, School of Medicine, Sir Run Run Shaw Hospital, Zhejiang University, Hangzhou, China

**Keywords:** Crohn’s disease, Phase angle, Bioelectrical impedance analysis, Postoperative complications, Ileocolic resection, Nutritional assessment

## Abstract

**Purpose:**

To investigate the association between preoperative phase angle (PhA), measured by bioelectrical impedance analysis, and short-term postoperative complications in patients with Crohn’s disease (CD) undergoing ileocolic resection.

**Methods:**

This retrospective cohort study included consecutive patients with CD who underwent ileocolic resection between April 2021 and December 2024. Baseline demographic, clinical, and nutritional data were analyzed. Univariable and multivariable logistic regression models were employed to identify predictors of postoperative complications. The discriminative ability of PhA was evaluated using receiver operating characteristic (ROC) curve analysis, with additional stratification by sex.

**Results:**

Among 119 patients (median age 28 years; 72.3% male; median BMI 18.0 kg/m^2^), 25 (21.0%) experienced postoperative complications. Mean preoperative PhA was significantly lower in patients with complications compared with those without (4.1 ± 0.5° vs 4.8 ± 0.7°, *P* < 0.001). In multivariable analysis, higher preoperative PhA was independently associated with reduced odds of complications (OR = 0.203, 95% CI = 0.085–0.487, *P* < 0.001), whereas elevated C-reactive protein on postoperative day 3 was associated with increased odds (OR = 1.017, 95% CI = 1.007–1.028, *P* = 0.002). PhA demonstrated good overall discrimination (AUC 0.772, 95% CI 0.657–0.863). Sex-stratified analysis revealed superior discrimination in females (AUC 0.864, 95% CI 0.689–1.000; cut-off 3.9°) compared with males (AUC 0.748, 95% CI 0.625–0.857; cut-off 4.5°).

**Conclusion:**

Lower preoperative PhA values were associated with a higher risk of short‑term postoperative complications after ileocolic resection for CD. Findings support the potential incorporation of PhA into preoperative risk assessment to help identify higher‑risk patients and guide perioperative optimization.

**Supplementary Information:**

The online version contains supplementary material available at 10.1007/s00384-025-05053-y.

## Introduction

Crohn’s disease (CD) is a chronic, relapsing subtype of inflammatory bowel disease (IBD) that can affect any segment of the gastrointestinal tract. Despite advances in therapy, CD remains incurable, and patients often endure recurrent flares, progressive intestinal injury, and cumulative organ damage [1]. Although biologic agents have revolutionized the control of mucosal inflammation, many patients experience persistent disease activity and impaired gastrointestinal function. These manifestations often necessitate repeated bowel resections and lead to clinical sequelae such as malabsorption, chronic diarrhea, anorexia, and hypermetabolism. Collectively, these factors place individuals with CD at high risk for malnutrition and sarcopenia [2].

Nutritional status in CD is influenced not only by overt disease activity but also by disease duration and underlying subclinical inflammation. Consequently, malnutrition risk persists even during periods of apparent clinical remission [3]. In the perioperative setting, optimizing nutritional status is paramount, as robust evidence demonstrates that nutritional support—whether enteral or parenteral—improves postoperative recovery and clinical outcomes [4]. Therefore, there is an urgent need for reliable, valid, and practical tools to accurately assess nutritional risk in patients with CD preparing for surgery.

Conventional nutritional markers—including body mass index (BMI), serum albumin (Alb), and the Controlling Nutritional Status (CONUT) score—are routinely used for preoperative prognostication in CD [5, 6]. However, these indicators have significant limitations, as they often fail to capture specific alterations in body composition or cellular health. The limitations of static metrics have shifted focus toward more integrative and dynamic markers of nutritional status [7].

Bioelectrical impedance analysis (BIA) is a noninvasive, portable, and cost-effective technique that provides a quantitative assessment of body composition, including fat-free mass (FFM) and fat mass (FM) [8]. Among BIA-derived parameters, phase angle (PhA) has emerged as a critical indicator of cellular membrane integrity and body cell mass. High PhA values reflect preserved cellular function, whereas reduced values characterize catabolic states—such as malnutrition, sarcopenia, and cachexia—resulting from loss of cellular mass and altered membrane potential [9, 10]. Importantly, PhA has demonstrated sensitivity as a prognostic marker across various clinical contexts, where lower values predict adverse outcomes and higher values correlate with improved survival [11]. Thus, PhA offers potential utility not only for risk stratification but also as a guide for targeted nutritional intervention.

Patients with CD are particularly vulnerable to body composition alterations that independently predict adverse postoperative outcomes. While bioimpedance parameters have been shown to correlate with biochemical markers and disease activity in IBD [12], evidence regarding the specific prognostic role of PhA in surgical CD patients remains limited. Therefore, this study aims to evaluate the utility of preoperative PhA as a predictor of short-term postoperative complications in patients with CD undergoing ileocolic resection. Our objective is to validate PhA as a practical, evidence-based tool for preoperative risk stratification in this high-risk population.

## Materials and methods

### Study design and population

This retrospective cohort study analyzed data from patients with CD who underwent elective ileocolic resection at the Department of General Surgery, Sir Run Run Shaw Hospital, Zhejiang University School of Medicine, between April 2021 and December 2024. All surgeries were performed by a single specialized surgical team. This study adheres to the STROBE (Strengthening the Reporting of Observational Studies in Epidemiology) guidelines; the completed checklist is provided in Supplementary Table S1.

The primary objective was to evaluate the association between preoperative bioimpedance-derived PhA and short-term postoperative complications. Secondary objectives included the assessment of perioperative biochemical markers (e.g., CRP) as predictors of postoperative outcomes.

### Inclusion and exclusion criteria

Inclusion criteria were (1) age 16–65 years; (2) confirmed diagnosis of CD based on European Crohn’s and Colitis Organisation (ECCO) guidelines; and (3) elective ileocolic resection. Exclusion criteria were (1) history of acute or chronic hepatic or renal insufficiency; (2) current dependency on parenteral nutrition; (3) presence of a stoma (ileostomy or colostomy) prior to the index surgery; (4) history of extensive small bowel resection (residual small intestine < 2 m); (5) pregnancy or lactation; (6) emergency surgery; (7) concomitant conditions significantly affecting body composition or fluid status (e.g., decompensated heart failure, untreated endocrine disorders); and (8) incomplete clinical or BIA data.

### Data collection and perioperative management

Data collection comprised demographic characteristics, disease duration, smoking status, medication history, nutritional support, and disease behavior (Montreal classification). Perioperative management followed a standardized institutional protocol. Systemic corticosteroids were tapered with the goal of discontinuation 4 weeks prior to surgery whenever clinically feasible. Biologic agents (e.g., anti-TNF-α) were scheduled such that the last dose was administered ≥ 4 weeks preoperatively, unless disease severity dictated otherwise.

### Bioelectrical impedance analysis (BIA)

Preoperative body composition was assessed using a multifrequency bioelectrical impedance analyzer (InBody 770, InBody Co., Ltd., Seoul, South Korea). Assessments were performed under standardized conditions: participants were barefoot, wore light clothing, and removed metallic objects. Measurements were taken with the subject standing upright on the platform, arms extended and abducted from the torso, and thighs separated to ensure no skin-to-skin contact. The device measures impedance at multiple frequencies across five body segments (right arm, left arm, trunk, right leg, left leg). Phase angle was calculated using the formula: PhA (degrees) = arctangent (Xc/R) × 180°/π, where Xc is reactance and R is resistance at 50 kHz. The mean of two consecutive measurements was used for analysis.

### Biochemical markers

Preoperative laboratory assessments included serum albumin, hemoglobin, and C-reactive protein (CRP). Postoperative monitoring was standardized: vital signs and drain output were recorded daily. Fasting blood samples were collected on postoperative days (POD) 1, 3, and 5 to quantify CRP, white blood cell count, hemoglobin, and albumin. POD 3 CRP was pre-specified as a key variable of interest based on its established utility in predicting anastomotic leakage and infectious complications [13, 14].

### Outcome definitions

The primary outcome was the incidence of postoperative complications within 30 days of surgery. Complications were graded according to the Clavien–Dindo classification [15]. Grades I–II were defined as minor complications, and grades III–IV as major complications. Outcomes were ascertained via electronic medical records review, covering the index hospitalization and any readmissions or outpatient visits within the 30-day postoperative period.

### Statistical analysis

Continuous variables were summarized as mean ± standard deviation (SD) or median (interquartile range [IQR]) as appropriate; categorical variables were summarized as counts and percentages. Group comparisons used Student’s *t*-test or Mann–Whitney *U* test for continuous variables and the chi-square or Fisher’s exact test for categorical variables. Univariable logistic regression identified variables associated with postoperative complications. Variables with *P* < 0.05 in univariable analysis were entered into a multivariable logistic regression to estimate adjusted odds ratios (ORs) with 95% confidence intervals (CIs). Discrimination of PhA was assessed by receiver operating characteristic (ROC) curves with area under the curve (AUC) and 95% CIs; the optimal cut‑off was determined by the Youden index. Sex‑stratified ROC analyses were conducted. All statistical tests were two-sided, with significance set at *P* < 0.05. Analyses were conducted using SPSS version 26.0 (IBM Corp., Armonk, NY, USA) and R version 4.3.0.

### Ethical considerations

The study was conducted in accordance with the Declaration of Helsinki and approved by the Ethics Committee of Sir Run Run Shaw Hospital, Zhejiang University School of Medicine (Approval No. 2023–630-01). Due to the retrospective nature of the study and the use of de-identified data, the requirement for written informed consent was waived.

## Results

### Baseline characteristics

A total of 119 patients with Crohn’s disease were included in the final analysis. The cohort consisted predominantly of males (72.3%, *n* = 86) with a median age of 28 years (IQR 22–39 years) and a median BMI of 18.0 kg/m^2^ (IQR 16.6–20.4 kg/m^2^). The median disease duration prior to surgery was 36 months (IQR 6–84 months). Detailed demographic and clinical characteristics are summarized in Table 1.

**Table 1:** Baseline characteristics of all the patients

Characteristics	**All (119)**	**Characteristics**	**All (119)**
Age#, year	28 (22–39)	Preoperative enteral nutrition, *n* (%)	52 (43.7)
Men, ***n*** (%)	86 (72.3)	Montreal classification, *n* (%)	
BMI^#^, kg/m^2^	18.0 (16.6–20.4)	Age, year	
Current smoking, ***n*** (%)	6 (5.0)	A1 (≤ 16)	3 (2.5)
Mean disease duration before surgery^#^, month	36 (6–84)	A2 (17–40)	89 (74.8)
Preoperative hemoglobin*, g/dL	12.2 ± 1.8	A3 (> 40)	27 (22.7)
Preoperative albumin*, g/L	38.5 ± 4.4	Location	
Preoperative C-reactive protein^#^, mg/L	4.6 (1.0–16.3)	L1 (ileal)	75 (63.0)
Preoperative phase angle*, °	4.6 ± 0.7	L2 (colonic)	9 (7.6)
Preoperative muscle mass*, kg	22.7 ± 4.6	L3 (ileocolonic)	35 (29.4)
Operative time*, min	162.4 ± 35.8	L4 (upper gastrointestinal)	0
Preoperative treatment, ***n*** (%)		Behavior	
5-ASA	12 (10.1)	B1 (inflammatory/failure of medical therapy)	1 (0.8)
Corticosteroids	3 (2.5)	B2 (stricturing)	63 (52.9)
Immunomodulators	10 (8.4)	B3 (penetrating)	34 (28.6)
Biologic therapy	57 (47.9)	B2 + B3	21 (17.6)
Others	37 (31.1)	Perianal disease	75 (63.0)

*5-ASA* 5-aminosalicylic acid

*Values are mean ± SD

#Values are median (interquartile range)

Regarding disease phenotype (Montreal classification), the majority were diagnosed between 17 and 40 years of age (A2, 74.8%) and exhibited ileal involvement (L1, 63.0%). Stricturing behavior (B2) was the most common phenotype (52.9%), followed by penetrating behavior (B3, 28.6%). Perianal disease was present in 63.0% of the cohort. Biologic therapy was the most frequently used preoperative medication (47.9%), and 43.7% of patients received preoperative enteral nutrition. In terms of nutritional and surgical parameters, the mean preoperative PhA was 4.6 ± 0.7°, and mean serum albumin was 38.5 ± 4.4 g/L. The median operative time was 162.4 ± 35.8 min.

### Postoperative complications

Postoperative complications occurred in 25 of 119 patients (21.0%) within 30 days of surgery (Table 2). Among these, 10 patients experienced minor complications (Clavien–Dindo grades I–II), with postoperative fever and transient bowel obstruction being the most frequent. Major complications (Clavien–Dindo grades III–IV) were identified in a subset of patients, primarily involving abdominopelvic collections (4.2%) requiring drainage. No mortality (grade V) was recorded during the study period.

**Table 2:** Postoperative complications for ileocolic resection in CD patients

Characteristics	**All (*n* = 119)**	**Characteristics**	**All (*n* = 119)**
Postoperative stay*, days	7 (7–8)	Major complications (grades III to IV)	15 (12.6)
Postoperative complications, ***n*** (%)	25 (21.0)	Gastrointestinal bleeding	3 (2.5)
Minor complications (grades I to II)	10 (8.4)	Anastomotic leakage	1 (0.8)
Fever > 38.5 °C after surgery	5 (4.2)	Abdominopelvic collection	5 (4.2)
Diarrhea	2 (1.7)	Pleural effusion	1 (0.8)
Early postoperative bowel obstruction	3 (2.5)	Intra-abdominal abscess	2 (1.7)
Postoperative blood transfusions	1 (0.8)	Intra-abdominal bleeding	3 (2.5)

*Values are median (interquartile range)

### Univariate analysis

Patients who developed postoperative complications had significantly lower preoperative PhA compared with those who did not (4.1 ± 0.5° vs 4.8 ± 0.7°, *P* < 0.001; Table 3). Furthermore, POD 3 CRP levels were significantly elevated in the complication group (median 126.9 mg/L [IQR 93.0–157.0] vs 80.3 mg/L [IQR 52.1–117.1], *P* < 0.001). Other baseline variables—including age, sex, BMI, disease duration, albumin, hemoglobin, and preoperative medication—did not differ significantly between the two groups (all *P* > 0.05).

**Table 3:** Univariate analysis of risk factors associated with postoperative complications

Characteristics	Postoperative complications (25)	No postoperative complications (94)	*P* value
Men, *n* (%)	19 (76.0)	67 (71.3)	0.828
BMI*, kg/m^2^	17.9 (16.7–20.1)	18.2 (16.6–20.5)	0.784
Current smoking, *n* (%)	0 (0.0)	6 (6.4)	0.341
Mean disease duration before surgery*, month	12.0 (4.0–60.0)	36.0 (7.0–84.0)	0.390
Preoperative hemoglobin*, g/dL	12.1 (11.4–13.6)	12.4 (11.4–13.5)	0.522
Preoperative albumin*, g/L	38.2 (34.3–42.9)	39.1 (36.9–41.2)	0.966
Preoperative C-reactive protein*, mg/L	3.9 (1.6–16.3)	4.6 (1.0–14.7)	0.715
Preoperative phase angle^#^, °	4.1 ± 0.5	4.8 ± 0.7	< 0.001
Preoperative muscle mass*, kg	21.9 (18.0–25.3)	22.9 (20.2–25.1)	0.144
Operative time^#^, min	173.4 ± 40.3	158.9 ± 38.2	0.135
Preoperative enteral nutrition, *n* (%)	11 (44.0)	41 (43.6)	0.973
Postoperative C-reactive protein on POD 3, mg/L	126.9 (93.0–157.0)	80.3 (52.1–117.1)	< 0.001
Montreal classification, *n* (%)			
Age, y			0.602
A1 (≤ 16)	0 (0.0)	3 (3.2)	
A2 (17–40)	20 (80.0)	69 (73.4)	
A3 (> 40)	5 (20.0)	22 (23.4)	
Location			0.535
L1 (ileal)	18 (72.0)	57 (60.6)	
L2 (colonic)	1 (4.0)	8 (8.5)	
L3 (ileocolonic)	6 (24.0)	29 (30.9)	
L4 (upper gastrointestinal)	0	0	
Behavior			0.057
B1 (inflammatory/failure of medical therapy)	1 (4.0)	0 (0.0)	
B2 (stricturing)	9 (36.0)	54 (57.4)	
B3 (penetrating)	9 (36.0)	26 (27.7)	
B2 + B3	8 (32.0)	14 (14.9)	
Perianal disease	7 (28.0)	54 (65.1)	0.213
Preoperative treatment, *n* (%)			0.659
5-ASA	2 (8.0)	10 (10.6)	
Corticosteroids	0 (0.0)	3 (3.2)	
Immunomodulators	2 (8.0)	8 (8.5)	
Biologic therapy	15 (60.0)	42 (44.7)	
Others	6 (24.0)	31 (33.0)	

*POD* postoperative day, *5-ASA* 5-aminosalicylicacid

*Values are median (interquartile range)

#Values are mean ± SD

### Multivariate analysis

In the multivariable logistic regression model (Table 4), preoperative PhA and POD 3 CRP remained independent predictors of postoperative complications. Higher preoperative PhA was associated with a significantly reduced risk of complications (OR = 0.203, 95% CI = 0.085–0.487, *P* < 0.001). This indicates that for every 1° decrease in PhA, the odds of complications increased approximately fivefold. Conversely, elevated POD 3 CRP was associated with increased odds of complications (OR = 1.017, 95% CI = 1.007–1.028, *P* = 0.002).

**Table 4:** Multivariate analysis of factors associated with postoperative complications

Characteristics	Multivariate		
	*P* value	OR	95% CI
Preoperative phase angle	< 0.001	0.203	0.085–0.487
Postoperative C-reactive protein on POD 3	0.002	1.017	1.007–1.028

*OR* odds ratio, *CI* confidence interval, *POD* postoperative day

### ROC curve analysis

**Fig. 1:**
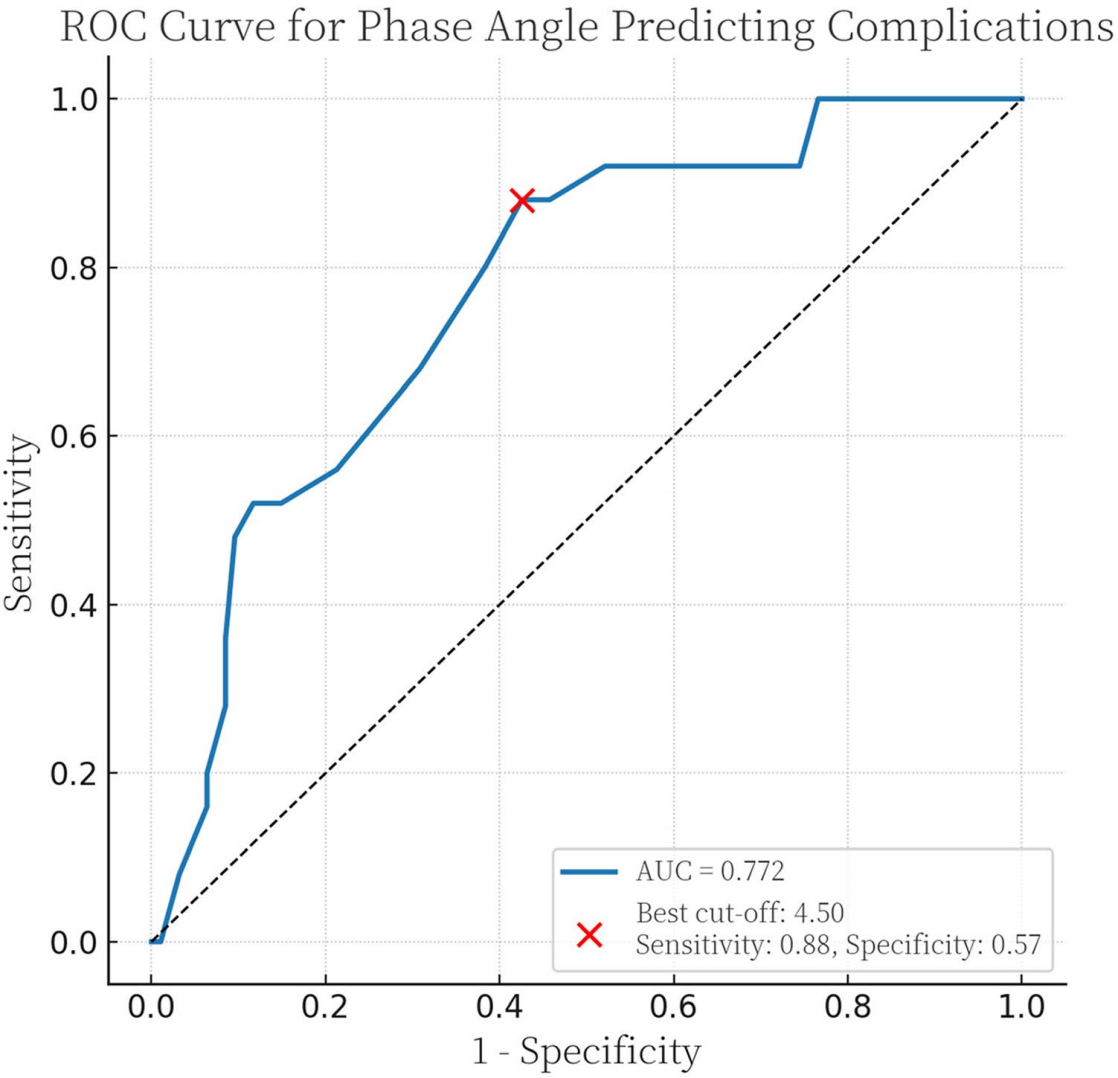
Receiver operating characteristic (ROC) curve analysis of preoperative phase angle (PhA) for predicting postoperative complications in the overall cohort (*n* = 119). The area under the curve (AUC) was 0.772 (95% CI 0.657–0.863), indicating good discriminative ability. An optimal cut-off value of 4.5° yielded a sensitivity of 88.0% and a specificity of 57.4%. The diagonal line represents the reference line of no discrimination (AUC = 0.5)

**Fig. 2:**
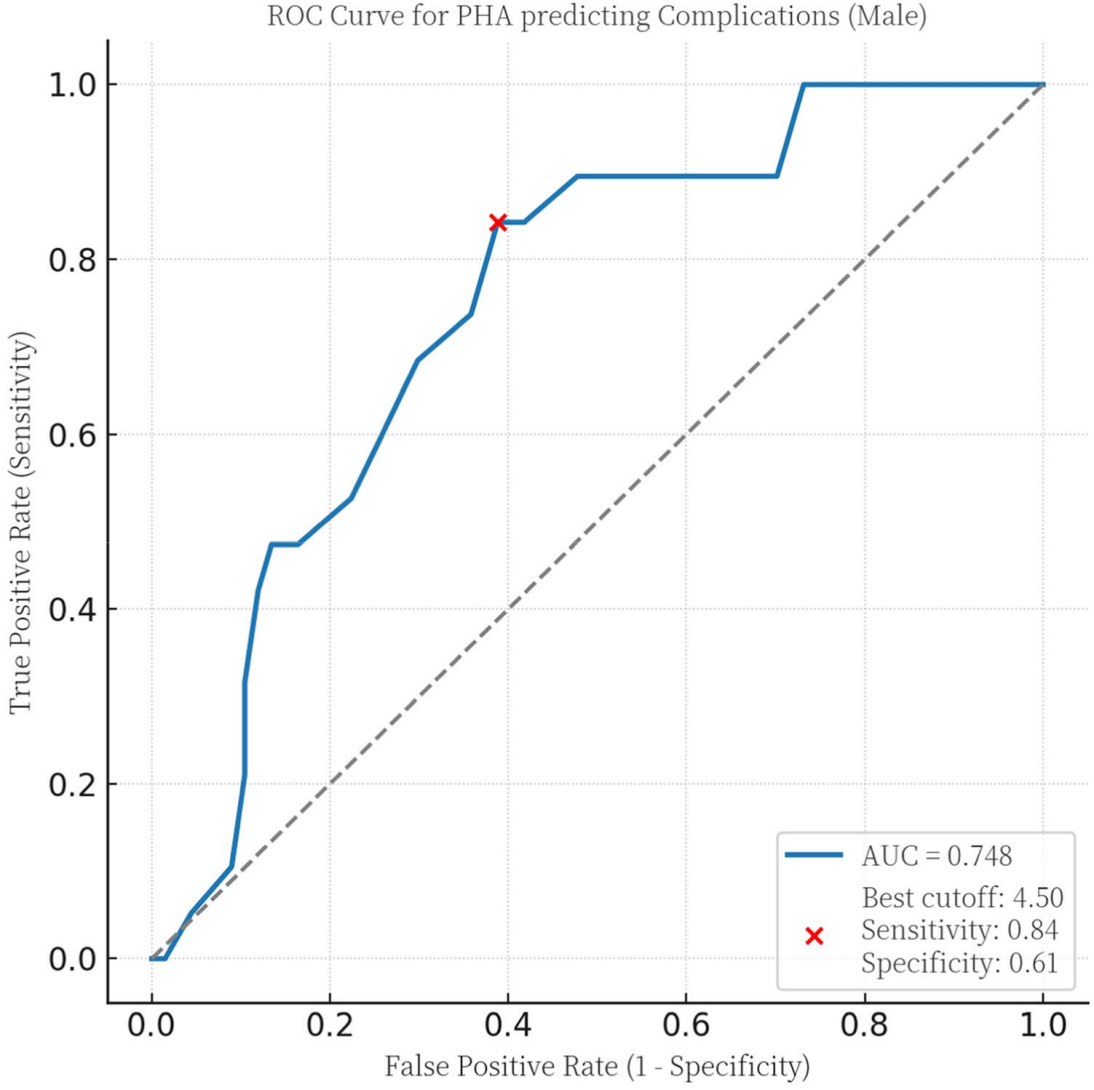
Receiver operating characteristic (ROC) curve analysis of preoperative PhA in the male subgroup (*n* = 86). The AUC was 0.748 (95% CI 0.625–0.857). The optimal cut-off value was identified at 4.5°, corresponding to a sensitivity of 84.2% and a specificity of 61.2%

**Fig. 3:**
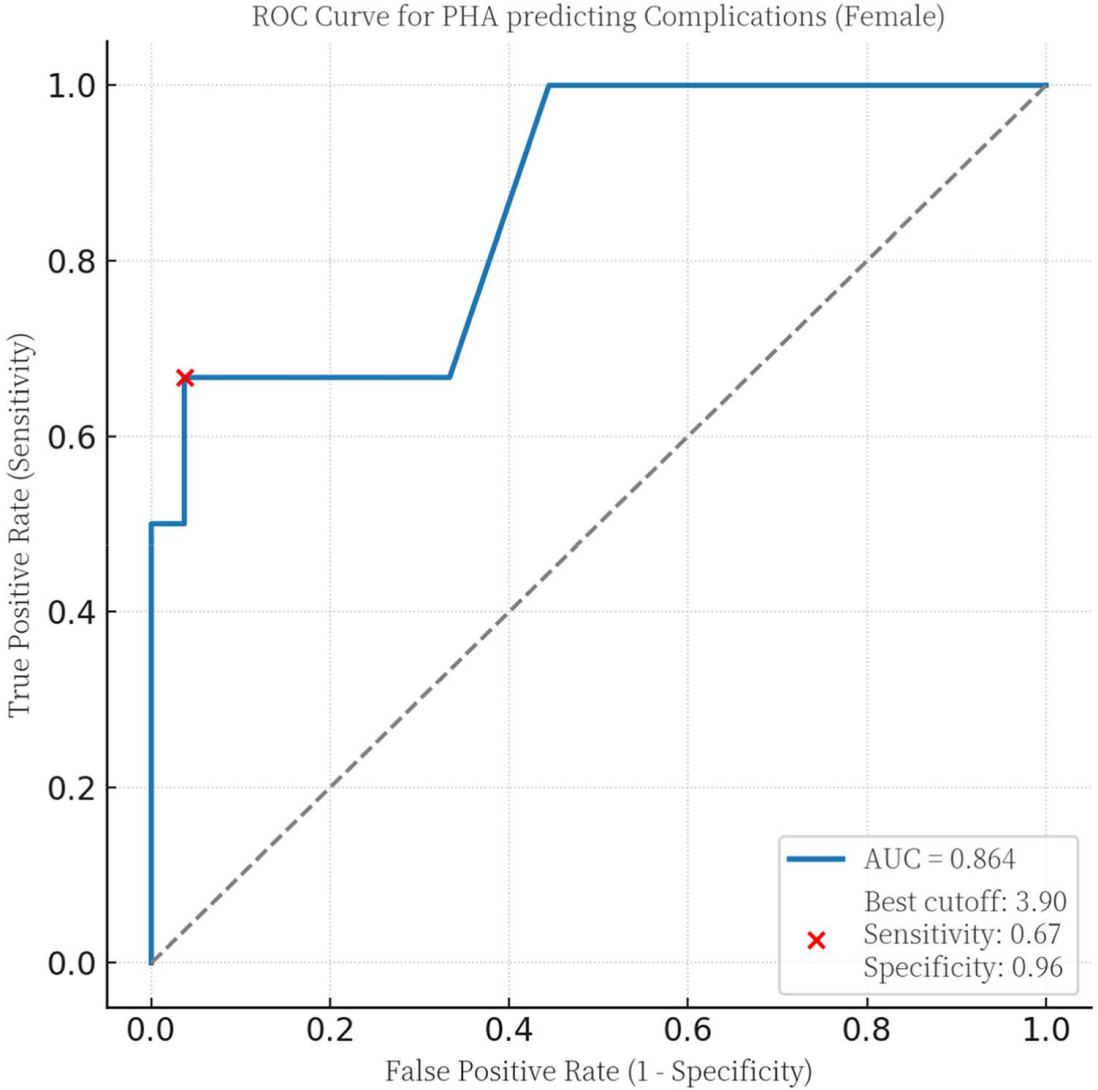
Receiver operating characteristic (ROC) curve analysis of preoperative PhA in the female subgroup (*n* = 33). PhA demonstrated excellent discriminative performance with an AUC of 0.864 (95% CI 0.689–1.000). The optimal cut-off value was 3.9°, with a sensitivity of 66.7% and a high specificity of 96.3%

ROC analysis demonstrated that preoperative PhA possessed good discriminative ability for predicting postoperative complications (AUC = 0.772, 95% CI = 0.657–0.863). The optimal cut-off value was determined to be 4.5°, yielding a sensitivity of 88.0% and specificity of 57.4% (Fig. 1). Sex-stratified analysis revealed differences in predictive performance. In male patients (*n* = 86), the AUC was 0.748 (95% CI = 0.625–0.857) with an optimal cut-off of 4.5° (Fig. 2). In female patients (*n* = 33), PhA demonstrated superior discriminative performance (AUC = 0.864, 95% CI = 0.689–1.000), with a distinct optimal cut-off of 3.9° (sensitivity 66.7%, specificity 96.3%) (Fig. 3). Comprehensive diagnostic performance metrics, including positive and negative predictive values, are presented in Table 5.

**Table 5:** ROC analysis of phase angle for predicting postoperative complications

Analysis	AUC	95% CI	*P*-value	Optimal cut-off (°)	Sensitivity (%)	Specificity (%)	PPV (%)	NPV (%)
Overall (*n* = 119)	0.772	0.657–0.863	< 0.001	4.5	88.0	57.4	31.4	95.6
Male (*n* = 86)	0.748	0.625–0.857	< 0.001	4.5	84.2	61.2	34.8	93.6
Female (*n* = 33)	0.864	0.689–1.000	0.002	3.9	66.7	96.3	80.0	92.9

*AUC* area under the curve, *CI* confidence interval, *PPV* positive predictive value, *NPV* negative predictive value

## Discussion

The present study provides the first evidence evaluating the prognostic utility of preoperative bioimpedance-derived PhA in patients with CD undergoing ileocolic resection. Our analysis of 119 consecutive patients demonstrates that lower preoperative PhA is an independent and robust predictor of short-term postoperative complications. Furthermore, we identified sex-specific PhA thresholds that enhance risk stratification, particularly in female patients. These findings suggest that PhA captures critical aspects of physiological resilience and cellular health that traditional nutritional markers may miss, positioning it as a valuable tool for preoperative optimization.

Optimizing surgical outcomes is a cornerstone of CD management, as complications can precipitate disease recurrence, impair quality of life, and necessitate further interventions. While current guidelines advocate for comprehensive preoperative nutritional assessment [16, 17], conventional indices such as BMI, serum albumin, and the CONUT score have significant limitations. BMI fails to distinguish between lean tissue and pathological fluid accumulation, while serum albumin is heavily influenced by the systemic inflammatory response typical of active CD, rendering it less reliable as a pure nutritional marker [18–21]. Consequently, there is a critical need for an integrative biomarker that reflects both nutritional status and inflammatory burden.

PhA has emerged as a clinically relevant indicator of cellular integrity and functional status across various chronic diseases [22]. Biologically, PhA reflects the ratio of reactance (cell membrane capacitance) to resistance (tissue hydration), thereby serving as a surrogate for body cell mass and membrane quality [23]. In the context of CD, where malnutrition affects 65–75% of patients due to malabsorption, catabolism, and chronic inflammation [24], PhA values are typically suppressed. Lower PhA values signify cytokine-mediated membrane damage and loss of metabolically active tissue [25, 27]. Therefore, PhA integrates the dual impact of malnutrition and inflammation, providing a biologically plausible index of the patient’s physiological reserve to withstand surgical stress.

In our cohort, multivariable analysis confirmed PhA as an independent protective factor (OR = 0.203), indicating that for every 1° decrease in PhA, the odds of complications increased approximately fivefold. This strong association persisted even after adjusting for confounders, whereas traditional markers like BMI and albumin did not retain significance. This aligns with findings by Cioffi et al. [28] and Zhang et al. [29], who reported that bioimpedance parameters are more sensitive than biochemical markers in detecting nutritional deterioration in IBD. Additionally, our study confirmed the value of POD 3 CRP as an independent predictor of complications, consistent with its established role in early detection of anastomotic leaks and infectious morbidity [30, 31]. The combination of preoperative PhA (to assess baseline reserve) and postoperative CRP (to monitor surgical stress) may offer a comprehensive strategy for perioperative risk management.

A novel finding of this study is the differential diagnostic performance of PhA across sexes. PhA demonstrated superior discriminative ability in females (AUC 0.864) compared with males (AUC 0.748), with distinct optimal cut-offs (3.9° vs. 4.5°). This discrepancy likely reflects physiological differences in body composition, as men typically possess greater muscle mass and body cell mass than women. The high specificity (96.3%) observed in females suggests that PhA is particularly effective at ruling in high-risk status in this subgroup. Future risk stratification models should account for these sex-specific differences to improve predictive accuracy.

To our knowledge, this is the first study to specifically investigate PhA as a predictor of surgical outcomes in CD. Our findings extend evidence from oncology and hepatopancreatobiliary surgery [32, 33] to the IBD population. Based on these results, we propose that PhA could be integrated into a practical clinical pathway: (i) routine nutritional screening (e.g., NRS-2002), followed by (ii) bedside PhA measurement to refine risk stratification, and (iii) targeted prehabilitation for patients falling below sex-specific thresholds (e.g., < 3.9° for females, < 4.5° for males). For these high-risk candidates, postponement of elective surgery to allow for intensive nutritional therapy—such as exclusive enteral nutrition—might be considered to improve cellular health and potentially reduce complication rates.

This study has several limitations. First, its retrospective, single-center design inherently introduces selection bias and limits generalizability. Second, although we adjusted for key covariates, residual confounding from unmeasured factors—such as precise cumulative corticosteroid dose or degree of mucosal inflammation—cannot be excluded. Third, while we identified significant associations, the observational nature of the study precludes causal inference. Finally, we focused on short-term complications; the impact of PhA on long-term outcomes, such as disease recurrence and quality of life, remains to be elucidated.

## Conclusion

Low preoperative phase angle is an independent predictor of short-term postoperative complications in patients with Crohn’s disease undergoing ileocolic resection. The identification of distinct sex-specific thresholds—3.9° for females and 4.5° for males—highlights the potential of PhA as a precise, individualized tool for risk stratification. These findings support the integration of bioimpedance analysis into routine preoperative assessment to facilitate the early identification of vulnerable patients. Future prospective, multicenter studies are warranted to validate these thresholds and determine whether PhA-guided prehabilitation strategies can effectively improve surgical outcomes in this high-risk population.

## Supplementary Information

Below is the link to the electronic supplementary material.

ESM 1 (DOCX 138 KB)

## Data Availability

Data are available from the corresponding authors upon reasonable request.
